# Molecular survey of cattle ticks in Burundi: First report on the presence of the invasive *Rhipicephalus microplus* tick

**DOI:** 10.1371/journal.pone.0261218

**Published:** 2021-12-10

**Authors:** Lionel Nyabongo, David O. Odongo, Gad Milton, Eunice Machuka, Patrick Vudriko, Roger Pelle, Esther G. Kanduma

**Affiliations:** 1 Faculty of Science and Technology, Department of Biology, University of Nairobi (UoN), Nairobi, Kenya; 2 Biosciences eastern and central Africa-International Livestock Research Institute (BecA-ILRI) Hub, Nairobi, Kenya; 3 National Veterinary Laboratory of Bujumbura, Bujumbura, Burundi; 4 Tick Unit, International Livestock Research Institute, Nairobi, Kenya; 5 College of Veterinary Medicine, Animal Resources and Biosecurity, Makerere University, Kampala, Uganda; 6 Faculty of Science and Technology, Department of Biochemistry, University of Nairobi, Nairobi, Kenya; Beni Suef University Faculty of Veterinary Medicine, EGYPT

## Abstract

A recent research study on prevalence of tick-borne pathogens in Burundi reported high prevalence and endemicity of *Theileria parva*, *Anaplasma marginale and Babesia bigemina* infections in cattle. Detailed information about tick species infesting animals, their distribution and genetic diversity in Burundi is outdated and limited. This study therefore assessed the prevalence and genetic diversity of tick species infesting cattle across agroecological zones (AEZs) in Burundi. A cross-sectional study on the occurrence of tick species was conducted in 24 districts of Burundi between October and December 2017. Differential identification and characterization of ticks collected was conducted using tick morphological keys and molecular tools (*cox*1 and 12S rRNA gene). Chi-square test was used to test for association between agroecological zones and the prevalence of tick species. Phylogenetic relationships were inferred using bayesian and maximum likelihood algorithms. A total of 483 ticks were collected from the five AEZs sampled. Six tick species comprising of *Rhipicephalus appendiculatus*, *R*. *sanguineus*, *R*. *evertsi evertsi*, *R*. *microplus*, *R*. *decoloratus and Amblyomma variegatum* were observed. *Rhipicephalus appendiculatus* were the most prevalent ticks (~45%). A total of 138 specimens (28%) were found to be *Rhipicephalus microplus*, suggesting an emerging threat for cattle farmers. Twelve *R*. *appendiculatus cox*1 haplotypes were obtained from 106 specimens that were sequenced. Two *cox*1 haplotypes of *R*. *microplus* which clustered into previously reported Clade A were observed. *Rhipicephalus sanguineus* and *R*. *evertsi evertsi* ticks, the vectors of numerous zoonotic pathogens, were collected from cattle, which constitute a high risk for public health. These findings reveal an overlapping distribution of tick vectors in Burundi. The design of ticks and tick-borne diseases control strategies should consider the distribution of different vectors across the AEZs particularly the presence of the highly invasive *R*. *microplus* tick in Burundi and the potential risk of introducing the pathogenic *Babesia bovis*.

## Introduction

Several Ixodid ticks are vectors of economically important livestock diseases in sub-Saharan Africa. *Theileria parva* which is transmitted by *R*. *appendiculatus* is the most prevalent and economically important tick transmitted pathogen in cattle in sub-Saharan Africa countries [1,2]. Other important pathogens including *B*. *bigemina* and *A*. *marginale* that are transmitted by *R*. *decoloratus* and *Amblyomma variegatum* ticks respectively, have recently been reported across all the AEZs of Burundi [1].

A previous study conducted between 1980 and 1983 reported the occurrence of 13 tick species infesting animals in Burundi including *R*. *appendiculatus*, *R*. *evertsi evertsi*, *R*. *compositus*, *R*. *lunulatus*, *R*. *muhsamae*, *R*. *hurti*, *R*. *jeanneli*, *R*. *pravus*, *A*. *variegatum*, *R*. *decoloratus*, *Haemaphysalis aciculifer*, *Hyalomma marginatum rufipes* and *Ixodes cavipalpis* [3]. *Rhipicephalus appendiculatus*, *A*. *variegatum* and *R*. *decoloratus* were then the most common ticks across the country, the remainder were represented by few specimens. It is expected that the abundance and spatial spread of these vectors will have altered thirty years later.

Although economically important cattle pathogens were recently found to be prevalent in majority of the AEZs in Burundi [1], the current occurrence and distribution of tick vectors transmitting them, and the economic losses caused by the ticks and tick-borne diseases (TTBDs) remain unknown. In neighboring Tanzania, direct and indirect losses due to TTBDs were estimated to approximatively 364 million USD annually [4]. Determining the extent and spatial spread of tick vectors and tick-borne diseases and their associated losses would aid in planning and allocation of resources for their control in Burundi.

Agro-ecological zones and livestock production systems have been shown to influence ticks and tick-borne diseases epidemiology in cattle in eastern African countries [5]. In Burundi, the traditional extensive agropastoral system has over the years been gradually replaced by mixed crop-livestock production systems, where farmers integrate livestock and traditional crop production [6]. Dynamic livestock systems and environmental characteristics in turn influence vector populations and pathogen prevalence and distribution [3]. For instance, *R*. *appendiculatus* abundance was relatively low in areas where the altitude exceeds 2300 meter or in the region with monomodal rainfall. In the lowland area, *R*. *appendiculatus* accomplished two life cycle per year while in the highland region, only one was possible [3]. The current range of distribution of this important vector is not known given that an expanding distribution range had been predicted in sub-Saharan Africa [7].

Tick control in Burundi is mainly achieved through the application of acaricides. In an attempt to reduce the cost of tick control for farmers while maintaining endemicity of tick transmitted diseases, seasonal application of acaricides was suggested in 1980s [8]. At that time, the period of treatment coincided with maximal tick feeding activities (4 months per year).

Highly productive cattle are now being imported from neighboring countries (Uganda and Tanzania) and distributed to farmers to increase cattle productivity [1]. These animals (Holstein and crossbreed cattle) are more susceptible to tick and tick-borne infections, with observed high mortality rates due to East Coast Fever (ECF). Farmers have adopted acaricide application twice weekly to prevent frequent infections [8]. Though the use of acaricide contributes to reduced tick infestation and tick-borne pathogen infections, the emergence of tick resistant populations has been reported [9,10]. Moreover, acaricides are costly for farmers and concern over chemical residues responsible for environment pollution. Alternative ecological viable control measures being suggested include the use of tick vaccine and rearing of genetic resistant animals [9]. Although tick vaccines are effective, cross protection is still limited between tick species [2]. The biological process in which cattle acquire genetic resistance against tick infestation is still unclear.

Data on tick species infesting animals, their occurrence and distribution in Burundi is outdated and limited. Furthermore, tick identification during an earlier study [3] conducted in the early 80s was based on phenotypic characteristics only. Recently, *R*. *appendiculatus* molecular data was generated based on *cox*1 gene but was limited to two AEZs of Burundi [11]. Estimates of tick dispersal, phylogenetic structure, gene flow, mating patterns and evolutionary process may have critical implications in estimating tick transmitted pathogens dispersal, pathogen transmission mechanism and host-vector interaction [12]. Therefore, the objective of this study was to determine the range distribution and genetic characterization of tick species infesting cattle across 5 AEZs of Burundi. This information will guide the design of effective and strategic ticks and tick-borne diseases control measures.

## Methods

### Ethical statement

This study was approved by the Directorate of Animal Health, Ministry of Agriculture and Livestock of Burundi on July 13, 2016. Verbal consent was obtained from cattle owners at the time of the farm visit.

### Study area, design, tick collection and data analysis

The study was carried out in Burundi between October and December 2017. The location, the climate, the vegetation, and the farming systems of the study area have been previously described [1]. Ticks were collected from 506 cattle during a cross-sectional study conducted in 24 districts across the 5 AEZs of Burundi. Detailed description of sampling strategy of cattle from which the ticks were collected was described in Nyabongo et *al*. [1].

Cattle were examined throughout the body for the presence of ticks. Infesting ticks were plucked from individual cattle using steel forceps and kept in labelled plastic vials containing 70% ethanol. Geographical coordinates and names of the localities were recorded for each sampling site. Tick samples were transported to the laboratory and kept at 4°C.

Morphological identification of ticks was performed at the Tick Unit (International Livestock Research Institute (ILRI), Nairobi, Kenya) using a stereo microscope with 100X magnification based on taxonomic keys [13]. Data were recorded in Microsoft Excel and chi-square test of independence was performed in R statistical software v3.5 to test the association between AEZs and tick species prevalence. Furthermore, a subset of 226 ticks of different species were randomly selected from the pool of ticks for molecular characterization.

The R package named “rentrez” was used to search the GenBank database and download available reference *R*. *appendiculatus cox*1 from Kenya, Republic Democratic of Congo and Uganda to allow comparison with Burundian *cox*1 sequences generated in the present study.

### DNA extraction and PCR amplification

Genomic DNA was extracted from the ticks using the DNeasy Blood & Tissue kit (Qiagen, Hilden, Germany) following the manufacturer protocol and the DNA stored at -20°C for further genetic analysis. Two mitochondrial genes (*cox*1 and 12S rRNA) were targeted for molecular characterization as described previously [14,15]. PCR products were purified using the QIAquick PCR Purification Kit (Qiagen, Hilden, Germany) following the manufacturer’s protocol. The purified products were sequenced at Macrogen (Macrogen Europe B.V., the Netherlands) using respective gene primers (reverse and forward) used for the PCR amplification.

### Molecular data analysis

The CLC Main Workbench 7.7 (Qiagen, Hilden, Germany) was used to assemble and manually edit DNA sequences. They were trimmed to remove low quality reads. The same program was used to translate the *cox*1 consensus sequences into protein sequences. Molecular identification of tick species was conducted by comparing the sequences from this study with those available in NCBI database using the BLASTn algorithm (http://blast.ncbi.nlm.nih.gov/Blast.cgi). The DNA sequences of each gene were aligned using MEGA 7 software (https://www.megasoftware.net/) by applying the ClustalW algorithm and the aligned sequence exported as FASTA file. The aligned datasets were imported into the DnaSP 6.12 software (http://www.ub.edu/dnasp/) to compute the number of haplotypes (H) and the haplotype diversity (Hd). Synonymous and non-synonymous substitutions were also calculated.

Molecular phylogenetic tree of the *cox*1 sequences was constructed using Bayesian methods. Likelihood scores were computed in the jModelTest 2.1 software and the best evolutionary model was selected (HKY+I+G) based on hierarchical likelihood ratio test (hLRT), Bayesian information criterion (BIC) and the Akaike information criterion (AIC). Bayesian analyses were performed using BEAST software. Markov chains were run for 10^7^ generations and trees were sampled over 1000^th^ iteration. Consensus tree was generated using a burnin of 25% and a posterior probability limit of 50%. The bayesian tree generated was imported into the ITOL online software (https://itol.embl.de/) for displaying and annotation. A Maximum Likelihood (ML) tree was also generated in MEGA 7 and branch support was assessed by bootstrap analysis with 1000 replicates.

## Results

### Ticks identification, prevalence and distribution across AEZs

A total of 483 ticks were collected across the five AEZs sampled. The tick species identified, and their prevalence are summarized in Table 1. *R*. *appendiculatus* and *R*. *microplus* were the most prevalent compared to other tick species (with 44.9 and 28.5% respectively). Morphological identification of Burundian *R*. *microplus* specimen was based on the distinguishing features of the species as illustrated in Walker *et al*. [13]. The adult female presented a hypostomal teeth arranged in a typical 4+4 arrangement on the ventral view. The concavity on article one of the palpal segment had no setae. The coxae I showed distinct internal and external spurs. The genital aperture had a broad "U" shape between coxae I and II. The basis capituli on the dorsal view had a distinct cornua. The Burundian *R*. *microplus* specimen were further characterized and confirmed using *cox*1 and 12S rRNA genes. This is the first record of *R*. *microplus* in Burundi.

**Table 1:** Prevalence and distribution of tick species across AEZs.

		*R*. *appendiculatus*			*A*. *variegatum*			*R*. *decoloratus*			*R*. *microplus*			*R*. *sanguineus*			*R*. *evertsi evertsi*		
AEZ	No. ticks sampled	n	Prev** (%)	95% CI	n	Prev (%)	95% CI	n	Prev (%)	95% CI	n	Prev (%)	95% CI	n	Prev (%)	95% CI	n	Prev (%)	95% CI
CND*	114	74	64.91	55.79–73.05	13	11.4	6.78–18.53	10	8.77	4.83–15.39	13	11.4	6.78–18.53	1	0.88	0.15–4.8	3	2.63	0.89–7.45
Depressions	94	22	23.4	15.99–32.9	26	27.66	19.63–37.44	0	0	0	42	44.68	35.03–54.74	4	4.26	1.66–10.43	0	0	0
Highlands	78	30	38.46	28.44–49.55	12	15.38	9.02–24.99	1	1.28	0.22–6.91	35	44.87	34.33–55.89	0	0	0	0	0	0
Imbo	87	60	68.97	58.61–77.71	5	5.75	2.47–12.75	1	1.15	0.2–6.22	11	12.64	7.2–21.23	8	9.2	4.73–17.1	2	2.3	0.63–7.99
Slope CND	110	31	28.18	20.62–37.21	30	27.27	19.82–36.25	9	8.18	4.36–14.82	37	33.64	25.49–42.88	0	0	0	3	2.73	0.93–7.71
Total	483	217	44.93	40.54–49.38	86	17.81	14.65–21.46	21	4.35	2.86–6.55	138	28.57	24.72–32.75	13	2.69	1.57–4.55	8	1.66	0.84–3.23

* Congo Nile Divide.

**Prevalence.

*Rhipicephalus appendiculatus* were most frequent in the CND and Imbo AEZs while *R*. *microplus* were mostly encountered in the Central highlands (Table 1). On the contrary, the proportion of *R*. *decoloratus*, *R*. *sanguineus* and *R*. *evertsi evertsi* ticks were low (< 5%). The Pearson Chi-square test showed that tick species prevalence was statistically associated with AEZs (*χ*^2^  = _ _137.66, *df*  =  20, *P* < 0.0001).

Molecular characterization was conducted by sequencing of the *cox*1 and 12S rRNA genes. Nucleotide BLAST analysis of both *cox*1 and 12S rRNA sequences showed a high identity value with sequences available in GenBank database. Percentage of similarity for *R*. *microplus*, *R*. *appendiculatus*, *R*. *decoloratus*, *A*. *variegatum* and *R*. *evertsi evertsi* ticks ranged from 99.84 to 100%, 99.17 to 100%, 98.64 to 100%, 96.74 to 100%, 98.16 to 100% respectively (S1 Table). Dogs are the preferred host of *R*. *sanguineus*; therefore, the brown dog tick was excluded from further genetic analysis.

### Genetic diversity and haplotype frequency

Analysis of the Burundian *cox*1 sequences revealed highest haplotype diversity in *R*. *decoloratus* (0.848±0.032), *R*. *appendiculatus* (0.737±0.035) and *A*. *variegatum* (0.629±0.078) sequences while the lowest diversity was observed in *R*. *evertsi evertsi* (0.333±0.215) and *R*. *microplus* (0.050±0.047) (S2 Table). Analysis of the 12S gene sequences displayed similar results. All the 34 *cox*1 and 18 12S rRNA haplotype sequences generated in the present study were deposited in GenBank database under accession numbers MZ356568-MZ356569 for *R*. *microplus cox*1 haplotypes, MZ356570-MZ356581 for *R*. *appendiculatus*, MZ356905-MZ356916 for *R*. *decoloratus*, MZ356919-MZ356924 for *A*. *variegatum* and MZ357052-MZ357053 for *R*. *evertsi evertsi* haplotypes. GeneBank accession numbers provided for *R*. *microplus 12S* rRNA haplotype are MZ361320, MZ361313-MZ361319 for *R*. *appendiculatus*, MZ361310-MZ361312 for *R*. *decoloratus*, MZ361303- MZ361308 for *A*. *variegatum* and MZ361309 for *R*. *evertsi evertsi* haplotypes.

Comparison of the Burundian *R*. *appendiculatus* haplotypes generated in the present study with characterized haplotypes from the region showed a higher haplotype diversity (Hd) of between 0.737 and 0.978, with a nucleotide diversity (Pi) of between 0.0056 and 0.0169 (Table 2) compared to Hd of between 0.72 and 0.77 and Pi of 0.003 as reported in an earlier study [11].

**Table 2:** Mitochondrial nucleotide and haplotype diversity of Burundian *R*. *appendiculatus cox*1 haplotypes and other reference haplotypes from East and Central Africa described in earlier studies.

Country	N	S	H	Hd ± SD	Pi ± SD	K	Theta (per site)	Theta (sequence)
Burundi	106	20	12	0.737±0.035	0.00568±0.001	3.045	0.00713	3.82
DRC	52	27	22	0.962±0.009	0.01093±0.00138	5.856	0.01115	5.975
Kenya	99	30	28	0.947±0.009	0.01367±0.00059	7.328	0.01083	5.806
Uganda	10	21	9	0.978±0.054	0.01692±0.00180	9.067	0.01385	7.423
Overall	267	51	51	0.915±0.010	0.0108±0.00061	5.827	0.01544	8.276

N: Sample size, S: Number of polymorphic sites; H: Number of Haplotypes; Hd: Haplotype (gene) diversity, SD: standard deviation, Theta: Watterson estimator (from S). K: Average number of nucleotide differences, Pi: Nucleotide diversity.

A total of twelve haplotypes were obtained from 106 Burundian *R*. *appendiculatus cox*1 sequences analysed. Haplotype H1 had the highest frequency (45.28%, 48/106); followed by H2, H3, H4, H6, H9 which accounted for 20.75% (22/106), 11.32% (12/106), 5.66% (6/106), 5.66% (6/106) and 2.83% (3/106) respectively. Haplotypes H7, H10, H11 were observed twice while the haplotypes H5, H8, H12 were singletons. Two haplotypes were observed in *R*. *microplus* with haplotype H1 being the most common (97.5%, 39/40), the remainder (H2) was observed once. Six haplotypes of *A*. *variegatum* were obtained with H2 being the most common (57.57%, 19/33), followed by the haplotypes H4 (18.18%, 6/33) and H1 (15.15%, 5/33). Haplotypes H3, H5, H6 were observed once. Twelve haplotypes of *R*. *decoloratus* were observed with haplotypes H10, H1 and H3 having the highest frequency with 27.90% (12/43), 23.25% (10/43) and 16.27% (7/43) respectively. Haplotypes H2, H4, H6, H9 and H11 were observed twice while H5, H7, H8 and H12 were observed once. Haplotype H1 of *R*. *evertsi evertsi* was the most common (83.33%, 5/6) while H2 was observed once.

Synonymous mutations were identified in most of the *R*. *appendiculatus cox*1 sequences, except for haplotype 5 which had a non-synonymous mutation at position 477 with reference to the GenBank *cox*1 sequence Acc. No. MF458950.1, yielding an amino acid substitution in the cytochrome oxidase protein sequence (change from an arginine to isoleucine residue).

Non-synonymous mutations were detected at position 502 of haplotype H5 of *R*. *decoloratus* and at six positions of haplotype H8, with reference to the *cox*1 sequence Acc. No KY678129.1. The non-synonymous mutations in *R*. *decoloratus* sequences yielded single amino acid substitution (change from leucine to methionine residue) for haplotype H5 and multiple substitutions of amino acid residues for haplotype H8 (change from alanine to proline residue, tryptophan to serine, leucine to serine and proline to arginine residue). All SNPs in the *cox*1 sequences of *R*. *microplus*, *A*. *variegatum* and *R*. *evertsi evertsi* ticks were synonymous, with no change of amino acid residue.

### Phylogenetic structure

**Fig 1:**
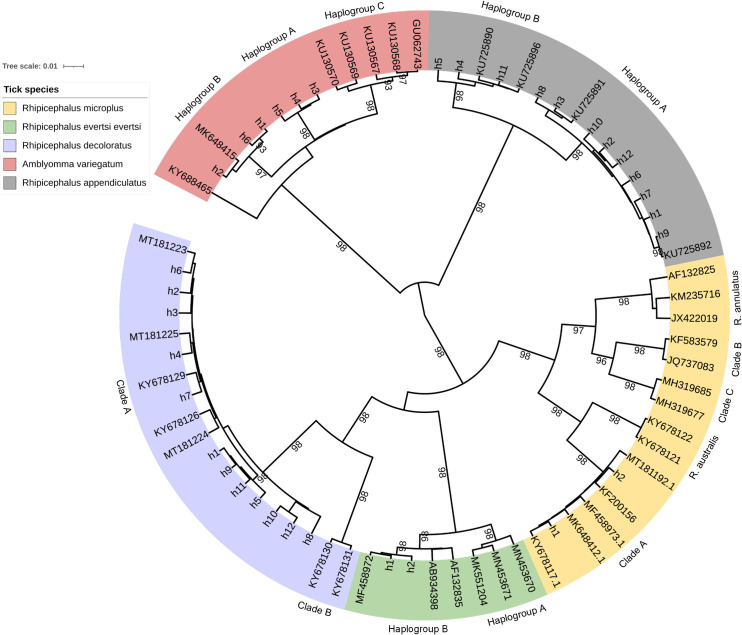
Bayesian phylogenetic tree based on *cox*1 gene. Haplotypes generated in the present study are labeled with a letter h followed by a number (h_1_, h_n_) while reference sequences from GenBank are represented by their accession numbers. Clades with a posterior probability > 95% are shown.

A Bayesian phylogenetic tree (Fig 1) was constructed based on 34 *cox*1 haplotypes (12 *R*. *appendiculatus*, 2 *R*. *microplus*, 12 *R*. *decoloratus*, 6 *A*. *variegatum* and 2 *R*. *evertsi evertsi*) combined with 34 reference *cox*1 sequences from GenBank (4 *R*. *appendiculatus*, 10 *R*. *microplus*, 7 *R*. *decoloratus*, 7 *A*. *variegatum* and 6 *R*. *evertsi evertsi*).

Burundian *R*. *appendiculatus* collected in the present study clustered into two well resolved haplogroups (HgA and HgB) with posterior probability of 100% (Figs 1 and S1). Haplogroup A consisted of haplotypes H1-H3, H6-H10 and H12 along with representative *cox*1 sequences (KU725891, KU725892) from Kenya. Haplotypes H4, H5 and H11 clustered in Haplogroup B together with *cox*1 sequences previously generated [15]. The within group mean genetic distance were 0.002 and 0.003 in haplogroup B and A of *R*. *appendiculatus* ticks respectively. The net between group mean distance (between haplogroup A and B) was 0.013.

*Amblyomma variegatum* sequences clustered into 3 groups (Haplogroup A, B and C) (Figs 1 and S1). Haplotypes H1, H2, H6, formed haplogroup B along with representative *A*. *variegatum cox*1 sequences from Cameroon (MK648415.1) and Uganda (KY688465.1). Haplogroup A contained only haplotypes collected in Burundi (H3, H4 and H5) while haplogroup C consisted of *A*. *variegatum cox*1 sequences from Nigeria (KU130569.1, KU130570.1) and Senegal (KU130567.1, KU130568.1, GU062743.1). The within group mean genetic distance were 0.001; 0.012 and 0.003 for *A*. *variegatum* haplogroup A, B, and C respectively. The net between group mean distances were 0.005; 0.017 and 0.016 between haplogroup A and B, A and C, B and C respectively.

*Rhipicephalus decoloratus* haplotypes clustered into two well resolved groups named Clade A and B (Figs 1 and S1). Haplotypes generated in the present study clustered into Clade A along with representative sequences from South Africa (KY678129), Burkina Faso (KY678126), Cameroon (MT181224, MT1812245) and Kenya (MT181223). Two *R*. *decoloratus cox*1 sequences from South Africa (KY678130, KY678131) formed a genetically distant group (Clade B). The within group mean genetic distance was 0.004 in Clade A while two sequences from South Africa which formed Clade B had a mean genetic distance of 0.001. The net between group mean distance (between Clade A and B of *R*. *decoloratus*) was 0.053.

*Rhipicephalus microplus* has five known clades namely Clade A (African, Asian and Latin America), B (Chinese), C (Malaysian), *R*. *annulatus* and *R*. *australis* [16,17]. Burundian haplotype H1 and H2 generated in the present study clustered in Clade A together with African reference sequences from Cameroon (MK648412), Kenya (MT181192), DRC (MF458973) and South Africa (KY678117) supported by a posterior probability of 97% (Figs 1 and S1). The within group mean genetic distances were 0.001; 0.002; 0.007; 0.000001; 0.001 in Clade B, C, *R*. *annulatus*, *R*. *australis* and Clade A, respectively. With reference to Clade A which includes Burundian haplotypes, the net between group mean distances were 0.046; 0.50; 0.36 and 0.32 for the clade B, C, *R*. *annulatus* and *R*. *australis*, respectively.

*Rhipicephalus evertsi evertsi* haplotypes formed two well resolved groups (Haplogroup A and B). Haplogroup B consisted of haplotypes H1 and H2 collected in Burundi along with reference *cox*1 sequences from DRC (MF458972) and Uganda (AB934398). Haplogroup A was formed by representative *cox*1 sequences from Lesotho (MN453670, MN453671, MK551204). The within group mean genetic distance was 0.005 and 0.007 in *R*. *evertsi evertsi* Haplogroup A and B respectively. The net between group mean distance (between Haplogroup A and B) was 0.016.

The phylogenetic relationship revealed by the ML tree (S1 Fig) was similar to the Bayesian tree. The sister ticks, *R*. *microplus* and *R*. *decoloratus* formed a paraphyletic group and shared a common ancestor with the two-host tick, *R*. *evertsi evertsi*.

Eighteen haplotypes of 12S rRNA were observed from the 210 sequenced specimens analysed in the present study (7 *R*. *appendiculatus*, 1 *R*. *microplus*, 3 *R*. *decoloratus*, 6 *A*. *variegatum* and 1 *R*. *evertsi evertsi*) combined with 38 reference 12S rDNA haplotypes (10 *R*. *appendiculatus*, 13 R. *microplus*, 3 *R*. *decoloratus*, 7 *A*. *variegatum* and 5 *R*. *evertsi evertsi*) retrieved from GenBank. The ML tree generated revealed a phylogenetic structure similar to the *cox*1 tree, with haplotypes clustering into two haplogroups for *R*. *appendiculatus*, *R*. *evertsi evertsi*, *R*. *decoloratus* and *A*. *variegatum* ticks. The single 12S rRNA haplotype of *R*. *microplus* generated in the present study along with 13 sequences retrieved from GenBank clustered into 5 groups (bootstrap value > 95%) (S2 Fig).

### Intensity of tick infestation, farmers practices and perceptions on the acaricide efficacy

The percentage of infested animals per AEZ and the intensity of infestation per animal are summarized in Table 3. The average number of ticks per animal across AEZs was 6.16. Tick infestation was high (12.2) in the slope of CND and the lowest was recorded in the CND (4.22). The proportion of infested cattle across AEZs was 15.42%. The highest proportion of infested cattle was observed in the Depressions AEZ (28.3%) and lowest in the Slope of CND (11.25%). *R*. *appendiculatus* infested relatively a higher number of cattle (6.52%); a lower proportion of infested cattle was recorded for *R*. *evertsi evertsi* (0.79%).

**Table 3:** Proportion of infested cattle and tick density across AEZs.

					Number of infested cattle per tick species												
					*R*. *appendiculatus*		*A*. *variegatum*		*R*. *decoloratus*		*R*. *microplus*		*R*. *sanguineus*		*R*. *evertsi evertsi*		
AEZ	No of sampled cattle	No of infested cattle	% of infested cattle	No Ticks	n	%	N	%	n	%	n	%	N	%	n	%	Tick infestation per animal
CND	166	27	16.27	114	15	9.04	3	1.81	6	3.61	3	1.81	1	0.60	1	0.60	4.22
Depressions	53	15	28.30	94	4	7.55	3	5.66	0	0	7	13.21	2	3.77	0	0	6.27
Highlands	107	15	14.02	78	6	5.61	6	5.61	1	0.93	3	2.80	0	0	0	0	5.20
Imbo	100	12	12.00	87	5	5.00	3	3.00	1	1.00	1	1.00	2	2.00	1	1.00	7.25
Slope CND	80	9	11.25	110	3	3.75	1	1.25	4	5.00	3	3.75	0	0	2	2.50	12.22
Total	506	78	15.42	483	33	6.52	16	3.16	12	2.37	17	3.36	5	0.99	4	0.79	6.19

Farmers practices on acaricide use and their perception on the level of efficacy are summarized in Table 4. A total of 13 commercial acaricide brands were recorded in the present study. Amidine molecules presented a higher number of brands available in the local market (9 out 13), followed by synthetic pyrethroids (3 out of 13) and organophosphate (1 out of 13). About 90% (348 out of 384) of farmers interviewed used amitraz, followed by synthetic pyrethroids which accounted for 8.5% (33 out of 384) of farmers while the least used acaricide was the organophosphate (n = 3). Within Amitraz products, three brands, Amitix, Amitraz and Norotraz were preferred by farmers and accounted for 28.13%, 20.57% and 15.36%respectively. Within the synthetic pyrethroids products, Cybadip was the most preferred being used by 8.07% (31 out of 384) of farmers. A high proportion of farmers interviewed in the present study applied acaricides once (59.38%, 228 out of 384) and twice a week (18.23%, 52 out of 384). The remainder applied the acaricide every two weeks (13.23%, n = 52) and once per month or between longer interval (7.29%, n = 28). About 60% (230 out of 384) of farmers rated the level of efficacy of acaricide as “Very Good”, and 35% (136 out of 384) as “Good”. The remainder were not happy with the acaricide efficacy and rated them as “Poor” (2.34%) and “Very poor” (1.30%).

**Table 4:** Type of acaricides used in the studied area and farmer’s perceptions on the level of efficacy.

								Frequency of application of the acaricide				Reported level of efficacy				
Brand name	Active ingredient	C*	Dilution**	No of farmers applying the acaricide	% within class	% Overall	No of Farmers who did not respond	twice a week	once a week	every two weeks	once per 4 weeks or longer interval	No of Farmers who did not respond	Very poor	Poor	Good	Very Good
Taktic	Amitraz	125	2:1	14	4.02	3.65	1	0	9	3	1	1	1	0	1	10
Ectraz	Amitraz	125	1:0.5	46	13.22	11.98	0	7	33	5	1	0	0	1	16	29
Amitix	Amitraz	125	2:1	108	31.03	28.13	0	21	59	19	9	0	2	1	50	55
Amitraz	Amitraz	125	2:1	79	22.70	20.57	4	10	46	9	10	2	0	7	22	48
Ashitraz	Amitraz	125	2:1	3	0.86	0.78	0	0	2	0	1	0	0	0	0	3
Intraz	Amitraz	125	2:1	8	2.30	2.08	0	0	2	6	0	0	0	0	8	0
Norotraz	Amitraz	125	2:1	59	16.95	15.36	0	5	41	9	4	0	2	0	22	35
Tixfix	Amitraz	125	2:1	15	4.31	3.91	0	10	5	0	0	0	0	0	0	15
Triatix	Amitraz	125	2:1	16	4.60	4.17	0	3	11	0	2	0	0	0	7	9
Cybadip	Cypermethrin	15	1:2	31	93.94	8.07	1	12	17	1	0	0	0	0	8	23
Cythrin	Cypermethrin	100	1.5:1	1	3.03	0.26	0	0	1	0	0	0	0	0	1	0
Deltathrin	Deltamethrin	25	1:1	1	3.03	0.26	0	0	1	0	0	0	0	0	1	0
Steladone	Chlorfenvinphos	300	1:1	3	100.00	0.78	0	2	1	0	0	0	0	0	0	3
Total				384			6	70	228	52	28	3	5	9	136	230

*Concentration: gramme/litre

** ml of acaricide/litre of water, AM: Amitraz, SP: Synthetic pyrethroid, OP: organophosphate.

## Discussion

Ticks infesting cattle have been reported in Africa with approximatively 40 tick species, both argasidae and ixodidae, having been recorded [13]. Their spatial distribution depends on various factors including vegetation cover, temperature, relative humidity, photoperiod and human activities [18]. Among the reported species, *R*. *appendiculatus* is the most prevalent in Eastern Africa although the invasive tick, *R*. *microplus* has been reported to be rapidly spreading across different African countries, replacing the indigenous *R*. *decoloratus* tick [19–21]. However, a recent study conducted in Cameroon (Central Africa), reported *A*. *variegatum* as the most prevalent tick [22].

The number of ticks per animal recorded in the present study is low with an average of 6 ticks per animal. An average tick density of 79 per animal in Serere district in Uganda has been reported [23]. Majority of farmers (96%) in Burundi apply acaricide for tick control [1]. The present study observed that 13 commercial acaricide brands are used in the studied area and about 95% of farmers rated their level of efficacy as “very good” and “good”, with 77% of farmers applying these chemicals once or twice weekly. Since the 1990’s, the government of Burundi through the Directorate General of Livestock, recommended the use of acaricide twice a week. This strategy led to the reduction of tick density per cattle. Tick seasonality could also explain the low tick infestation per animal recorded in the present study since the sampling was conducted in October when the climate is not suitable for tick development. A previous study suggested that maximal feeding activity is observed in the rainy season from February to May [8]. Burundian *R*. *appendiculatus* clustered with other East African ticks in two major lineages previously reported [15]. Similar results were found using the 12S rRNA gene. This is consistent with phylogenetic structure revealed by ML and neighbor joining algorithms from previous studies [11,15].

*Rhipicephalus appendiculatus*, which is the main vector of *T*. *parva* the causative agent of East Coast fever, is traditionally found at an altitude of up to 2000 and rainfall of between 500 and 2000 mm per year. It was the most prevalent tick (about 45%) distributed across all the AEZs of Burundi, although the Imbo and CND presented the highest proportion (more than 60%) compared to others AEZ analysed in this study. The tropical humid climate and the vegetation cover in Burundi offer a suitable environment for the tick species across the entire country. The life cycle of *R*. *appendiculatus* is sensitive to environmental conditions (humidity, vegetation, and altitude) and the presence of suitable animal hosts [24]. The hatching rate of tick eggs is influenced by the type of vegetation and level of humidity; at least 80% humidity is needed to avoid desiccation [3]. The presence of mammalian host is ultimately required for propagation of larval, nymphal and adult ticks. However, 50% of the *R*. *appendiculatus* adults can survive for as long as 17 months without feeding, while the larval and nymphal stages can survive from 2 to 9 months [3]. Cattle (host) are reared throughout the different AEZs of Burundi, enabling the vector to complete its life cycle and in doing so transmit *T*. *parva*. Human activities have also been implicated in the introduction of *R*. *appendiculatus* or its spatial expansion in Burundi [1]. Importation of infested cattle from Comoros islands led to the introduction and dispersal of the brown ear tick in areas where it had never been reported before, thus contributing to the emergence of the fatal ECF in naïve cattle population in the country [25]. A high haplotype diversity (Hd = 0.7) was found in Burundian *R*. *appendiculatus cox*1 sequences analysed which could be attributed to the extensive uncontrolled movement of cattle across national and international borders [1].

The invasive *R*. *microplus* tick has been spreading in many countries across Africa. Recent studies have confirmed the occurrence of the highly reproductive tick in Central (Cameroon) [20] and eastern Africa including Uganda and Kenya [21,23].

The present study identified and confirmed for the first time the presence of *R*. *microplus* in Burundi. A possible introduction route in Burundi is through transborder trade of infested cattle from neighboring countries such as Tanzania, Zambia and Uganda where *R*. *microplus* is known to occur. Since 1990’s, the government of Burundi imported highly productive cattle from Uganda and Tanzania. Those cattle were distributed to farmers in Burundi without any prior tick inspection and removal. In the current study, the proportion of this invasive tick was high in the central highlands as compared to other AEZs. The central highlands AEZ is characterized by an altitude between 1300–1500 meters, temperature between 18 and 21°C and rainfall between 1200 and 1600 mm per year. The Imbo zone and the Depressions AEZs which have a lower rainfall (<1000 mm per year) exhibited a relatively low proportion rate.

Ecological preferences of *R*. *microplus* in Central America have previously been described with stable temperature ranging between 20 and 25°C throughout the year being associated with high suitability index [26]. However, the minimum temperature required for African *R*. *microplus* is four degrees below the temperature required for American ticks [26,27]. The temperature range where *R*. *microplus* tick was recorded in Burundi was 18–21°C. Precipitation seasonality and relative humidity were also identified as important climate factors affecting *R*. *microplus* life cycle and tend to increase the number of tick generations and thus population dispersal [27,28]. *Rhipicephalus microplus* ticks require high rainfall although some variations were reported between season [27], although high proportion of ticks in areas of low rainfall between May and October in Africa have also been recorded [26]. On the contrary, high rainfall was associated with high proportion of *R*. *microplus* tick between November and March [27]. Moreover, uncontrolled movement of cattle within east and central Africa is suggested to contribute to the current dispersal and spread of *R*. *microplus* in the regions.

Recent studies reported a low genetic diversity of *R*. *microplus* circulating across Africa based on *cox*1 gene [20,21,23]. A recent observation showing the high similarity between the Kenyan *R*. *microplus cox*1 haplotypes to other African haplotypes which all clustered in Clade A has been reported [21]. The present study identified two haplotypes which also clustered in Clade A suggesting the existence of a highly similar genotype in Africa. The high prevalence of *R*. *microplus* and its spatial distribution in different AEZs of Burundi suggest an emerging threat of severe babesiosis to cattle production. In Tanzania and South Africa, *R*. *microplus* were reported to replace the indigenous *R*. *decoloratus* [19,29]. The tick is also highly prolific and cause substantial losses in cattle by exsanguination and by transmitting *Babesia bovis*, the causative agent of bovine babesiosis. It also has the capability of rapidly acquiring resistance against common acaricide molecules [30], thus making the presence of this tick in Burundi, an emerging threat to sustainable dairy farming.

The bont tropical tick *A*. *variegatum* is a three-host tick originating from Africa and the main vector of *Ehrlichia ruminantium* which causes heartwater and *Anaplasma marginale* which causes anaplasmosis in cattle. It is considered as the second most invasive tick species after *R*. *microplus* [31]. A high proportion of *A*. *variegatum* (>50%) occurred in the depressions and the slopes of Congo Nile Divide AEZs, which have an altitude ranging between 1000 and 1600 meters and annual rainfall ranging between 100 and 1300 mm. The proportion of *A*. *variegatum* were lower in the Imbo and CND AEZs (6 and 11% respectively) which are characterized by relatively extreme climatic conditions compared to other AEZs. Norval *et al*. [31] had recorded the presence of *A*. *variegatum* in the Zambezi valley and its lowland areas with altitude ranging between 900 and 1200 m above sea level. However, in Zimbabwe, a shift in spatial distribution of *A*. *variegatum* from their traditional foci (lowland area) which had a temperature of between 30 and 35°C and a rainfall of between 450 and 650 mm was reported [32].

Six haplotypes of *A*. *variegatum* were observed in Burundi with a high genetic diversity (Hd = 0.6). Burundian haplotypes clustered into 3 haplogroups (named A, B and C) along with other *cox*1 sequences retrieved from GenBank. Two genetic groups of *A*. *variegatum* (Western and Eastern Africa groups) in Africa with a low genetic diversity between them has been reported [33]. In the past years, *A*. *variegatum* spatial distribution has extended to the Caribbean and Indian Ocean islands from Africa [33]. The main factor which may be contributing to *A*. *variegatum* dispersal to geographically isolated islands is movement of animals through cattle trade and importation. The three genetic groups *of A*. *variegatum* observed in the present study were in sympatry in Burundi. Haplogroup A included only Burundian haplotypes (H2-H5) and clustered in well supported monophyletic group which was distinct from others haplogroups (B and C). Haplogroup C included only haplotypes from West Africa while a third group (named haplogroup B) included haplotypes from central and eastern African countries (Burundi, Uganda and Cameroon). These findings confirmed the phylogenetic structure of *A*. *variegatum* ticks as reported in previous studies (distinct western and eastern African populations) [33–35]. However, the eastern African population had two well resolved haplogroups.

*Rhipicephalus decoloratus* (the African Blue tick) are one host ticks transmitting *B*. *bigemina*, the causative agent of redwater in cattle. A low overall proportion (about 4%) of *R*. *decoloratus* was reported in the present study. However, *R*. *decoloratus* ticks were more prevalent in the CND and the surrounding slope areas which are characterized by relatively lower temperature (<20°C). The traditional habitat of *R*. *decoloratus* tick is a temperate climate with a grassland and wooded coverage [36]. Previous studies reported the presence of *R*. *decoloratus* across all the AEZs of Burundi. At that time, the African blue tick ranked as the second most prevalent tick after *R*. *appendiculatus* [3,37]. Displacement of the indigenous *R*. *decoloratus* by the invasive *R*. *microplus* has previously been observed in Tanzania, Cameroon and South Africa [19,20,29]. However, despite the rapid expansion capability of *R*. *microplus*, its dispersal could be restricted to the most suitable ecological niche [26].

Although *R*. *microplus*, *R*. *decoloratus* and *R*. *evertsi evertsi* display distinct biological (life cycle) parameters, the present study showed that they form a paraphyletic group and shared a common ancestor. Similar results previously reported [38,39], may indicate an African origin for this group of ticks.

A lower proportion rate of *R*. *evertsi evertsi* ticks was observed in the current study (about 2%). A high proportion of *R*. *evertsi evertsi* occurring in the traditional ecological niche, with an annual rainfall between 400 and 1000 mm had been reported in Senegal [35]. A suitable climate for the red legged tick exists in Burundi; however, the adult ticks prefer equids as host which are very few in number in the rural areas of the country. Thus, the absence/presence of the preferred animal host for *R*. *evertsi evertsi* may explain the variation in spatial distribution between regions which present similar climatic parameters.

Phylogenetic relationship of *R*. *evertsi evertsi* haplotypes generated in the present study revealed the existence of two well supported haplogroups. Haplogroup A consisted of haplotypes from Lesotho while haplotypes from Burundi, Uganda and DRC clustered into a separate haplogroup B. These findings may suggest that *R*. *evertsi evertsi* may be divided into two divergent and distinct populations, the eastern and southern Africa groups. However, due to the limited number of samples used in the present study, a comprehensive study is required to confirm the existence of the two groups. Although the proportion of the red legged tick is low in Burundi, it plays a role as a vector of *A*. *marginale* in cattle, and thus need to be taken into consideration by veterinary authorities when designing tick control strategies. Moreover, *R*. *evertsi evertsi* is known as a vector of *Rickettsia africae*, *R*. *conorii* and *Coxiella burnetii*, causative agents of African tick typhus, Mediterranean spotted fever and Q fever in humans, respectively [40], suggesting a threat for public health.

*Rhipicephalus sanguineus* tick was also found infesting cattle in Burundi. Though dogs are the preferred host, it occasionally feeds on cattle and human, transmitting zoonotic pathogens including *C*. *burnetii*, *Ehrlichia canis*, *R*. *conorii*, and *Rickettsia rickettsii* [41].

## Conclusion

A high prevalence of *R*. *appendiculatus* tick species was observed in Burundian cattle. Proportion of tick species was statistically associated with AEZs. Although all the studied tick species were found to occur in the 5 AEZs investigated, distinct suitable ecological niche for each tick species exists. *Rhipicephalus microplus* was recorded for the first time in Burundi and clustered in Clade A with sequences from other African countries. Further studies are needed to assess the efficacy of commonly used acaricides which could guide the design of an effective ticks and tick-borne diseases control program.

## Supporting information

S1 Fig Maximum likelihood phylogenetic tree based on *cox*1 gene. Haplotypes generated in the present study are highlighted with a red dot labeled with the letter h followed by a number (h_1_, h_n_). Evolutionary model used is Tamura 3-parameter (T92) with gamma distribution (+G = 4 categories). Clade with bootstrap value > 95% are shown.
(TIF)

S2 Fig Maximum likelihood phylogenetic tree based on 12S rRNA gene. Haplotypes generated in the present study are highlighted with a red dot. Evolutionary model used is HKY with gamma distribution (+G = 4 categories). Clade with bootstrap value > 95% are shown.
(TIF)

S1 Table Nucleotide blast results of tick haplotypes. (DOCX)

S2 Table Mitochondrial nucleotide and haplotype diversity of infesting cattle ticks in Burundi. (DOCX)

## Abbreviations

*cox*1: Cytochrome c Oxidase I
DNA: Deoxyribonucleic acid
ECF: East Coast fever
ILRI: International Livestock Research Institute
RNA: Ribonucleic acid
SNPs: Single nucleotide polymorphisms
TTBD: Ticks and Tick-Borne Disease

## References

[1] Nyabongo L, Kanduma EG, Bishop RP, et al. Prevalence of tick-transmitted pathogens in cattle reveals that *Theileria parva*, *Babesia bigemina* and *Anaplasma marginale* are endemic in Burundi. *Parasites and Vectors*. 2021;14(1). doi: 10.1186/s13071-020-04531-2 33402225 PMC7786990

[2] Nene V, Kiara H, Lacasta A, Pelle R, Svitek N, Steinaa L. The biology of *Theileria parva* and control of East Coast fever—Current status and future trends. *Ticks Tick Borne Dis*. 2016;7(4):549–564. doi: 10.1016/j.ttbdis.2016.02.001 26972687

[3] Kaiser MN, Sutherst RW, Bourne AS, Gorissen L, Floyd RB. Population dynamics of ticks on Ankole cattle in five ecological zones in Burundi and strategies for their control. *Prev Vet Med*. 1988;6(3):199–222. doi: 10.1016/0167-5877(88)90031-1

[4] Kivaria FM. Estimated direct economic costs associated with tick-borne diseases on cattle in Tanzania. *Trop Anim Health Prod*. 2006;38(4):291–299. doi: 10.1007/s11250-006-4181-2 17137131

[5] Gachohi J, Skilton R, Hansen F, Ngumi P, Kitala P. Epidemiology of East Coast fever (*Theileria parva* infection) in Kenya: past, present and the future. *Parasit Vectors*. 2012;5:194. doi: 10.1186/1756-3305-5-194 22958352 PMC3465218

[6] Manirakiza J, Hatungumukama G, Th Evenon S, et al. Effect of genetic European taurine ancestry on milk yield of Ankole-Holstein crossbred dairy cattle in mixed smallholders system of Burundi highlands. Published online 2017. doi: 10.1111/age.12578 28833335

[7] Olwoch JM, Van Jaarsveld AS, Scholtz CH, Horak IG. Climate change and the genus *rhipicephalus* (Acari: Ixodidae) in Africa. *Onderstepoort J Vet Res*. 2007;74(1):45–72. doi: 10.4102/ojvr.v74i1.139 17708153

[8] Moran MC, Nigarura G. Strategic tick control in Burundi. *Parassitologia*. 1990;32(1):177–184. Accessed February 12, 2019. http://www.ncbi.nlm.nih.gov/pubmed/2284129. 2284129

[9] Latif AA, Pegram RG. Naturally acquired host resistance in tick control in Africa. *Int J Trop Insect Sci*. 1992;13(04):505–513. doi: 10.1017/s1742758400016088

[10] Vudriko P, Okwee-Acai J, Tayebwa DS, et al. Emergence of multi-acaricide resistant *Rhipicephalus* ticks and its implication on chemical tick control in Uganda. *Parasit Vectors*. 2016;9(1):4. doi: 10.1186/s13071-015-1278-3 26727991 PMC4700616

[11] Amzati GS, Pelle R, Muhigwa J-BB, et al. Mitochondrial phylogeography and population structure of the cattle tick *Rhipicephalus appendiculatus* in the African Great Lakes region. *Parasit Vectors*. 2018;11(1):329. doi: 10.1186/s13071-018-2904-7 29855375 PMC5984310

[12] Araya-Anchetta A, Busch JD, Scoles GA, Wagner DM. Thirty years of tick population genetics: A comprehensive review. *Infect Genet Evol*. Published online 2015. doi: 10.1016/j.meegid.2014.11.008 25461844

[13] Walker A., Bouattour A, Camicas J., et *al*. *Ticks of Domestic Animals in Africa*: *A Guide to Identification of Species*.; 2003. Accessed October 27, 2020.

[14] Folmer O, Black M, Hoeh W, Lutz R, Vrijenhoek R. DNA primers for amplification of mitochondrial cytochrome c oxidase subunit I from diverse metazoan invertebrates. *Mol Mar Biol Biotechnol*. 1994;3(5):294–299. doi: 10.1071/ZO9660275 7881515

[15] Kanduma EG, Mwacharo JM, Githaka NW, et al. Analyses of mitochondrial genes reveal two sympatric but genetically divergent lineages of *Rhipicephalus appendiculatus* in Kenya. *Parasites and Vectors*. 2016;9(1). doi: 10.1186/s13071-016-1631-1 27334334 PMC4918217

[16] Burger TD, Shao R, Barker SC. Phylogenetic analysis of mitochondrial genome sequences indicates that the cattle tick, *Rhipicephalus (Boophilus) microplus*, contains a cryptic species. *Mol Phylogenet Evol*. 2014;76(1):241–253. doi: 10.1016/j.ympev.2014.03.017 24685498

[17] Low VL, Tay ST, Kho KL, et al. Molecular characterisation of the tick *Rhipicephalus microplus* in Malaysia: New insights into the cryptic diversity and distinct genetic assemblages throughout the world. *Parasites and Vectors*. 2015;8(1):341. doi: 10.1186/s13071-015-0956-5 26104478 PMC4482097

[18] Dantas-Torres F. Climate change, biodiversity, ticks and tick-borne diseases: The butterfly effect. *Int J Parasitol Parasites Wildl*. 2015;4(3):452–461. doi: 10.1016/j.ijppaw.2015.07.001 26835253 PMC4699983

[19] Lynen G, Zeman P, Bakuname C, et al. Shifts in the distributional ranges *of Boophilus ticks* in Tanzania: evidence that a parapatric boundary between *Boophilus microplus* and *B*. *decoloratus* follows climate gradients. *Exp Appl Acarol*. 2008;44(2):147–164. doi: 10.1007/s10493-008-9134-1 18266058

[20] Silatsa BA, Kuiate JR, Njiokou F, et al. A countrywide molecular survey leads to a seminal identification of the invasive cattle tick *Rhipicephalus (Boophilus) microplus* in Cameroon, a decade after it was reported in Cote d’Ivoire. *Ticks Tick Borne Dis*. 2019;10(3):585–593. doi: 10.1016/j.ttbdis.2019.02.002 30765191 PMC6446184

[21] Kanduma EG, Emery D, Githaka NW, Nguu EK, Bishop RP, Šlapeta J. Molecular evidence confirms occurrence of *Rhipicephalus microplus* Clade A in Kenya and sub-Saharan Africa. *Parasites and Vectors*. 2020;13(1):432. doi: 10.1186/s13071-020-04266-0 32854747 PMC7453536

[22] Silatsa BA, Simo G, Githaka N, et al. A comprehensive survey of the prevalence and spatial distribution of ticks infesting cattle in different agro-ecological zones of Cameroon. *Parasit Vectors*. 2019;12(1):489. doi: 10.1186/s13071-019-3738-7 31623642 PMC6796472

[23] Muhanguzi D, Byaruhanga J, Amanyire W, et al. Invasive cattle ticks in East Africa: Morphological and molecular confirmation of the presence of *Rhipicephalus microplus* in south-eastern Uganda. *Parasites and Vectors*. 2020;13(1). doi: 10.1186/s13071-020-04043-z 32245511 PMC7118885

[24] Madder M, Speybroeck N, Brandt J, Tirry L, Hodek I, Berkvens D. Geographic variation in diapause response of adult *Rhipicephalus appendiculatus* ticks. *Exp Appl Acarol*. 2002;27(3):209–221. doi: 10.1023/a:1021694207456 12593586

[25] Yssouf A, Lagadec E, Bakari A, et al. Colonization of Grande Comore Island by a lineage of *Rhipicephalus appendiculatus* ticks. *Parasites and Vectors*. 2011;4(1). doi: 10.1186/1756-3305-4-38 21414194 PMC3069959

[26] Estrada-Peña A, Bouattour A, Camicas JL, et al. The known distribution and ecological preferences of the tick subgenus *Boophilus* (Acari: *Ixodidae*) in Africa and Latin America. *Exp Appl Acarol*. 2006;38(2–3):219–235. doi: 10.1007/s10493-006-0003-5 16596355

[27] Estrada-Peñ A. Climate warming and changes in habitat suitability for *Boophilus microplus* (Acari: Ixodidae) in Central America. *J Parasitol*. 2001;87(5):978–987. doi: 10.1645/0022-3395(2001)087[0978:CWACIH]2.0.CO;2 11695419

[28] Marques R, Krüger RF, Peterson AT, De Melo LF, Vicenzi N, Jiménez-García D. Climate change implications for the distribution of the babesiosis and anaplasmosis tick vector, *Rhipicephalus (Boophilus) microplus*. *Vet Res*. 2020;51(1):81. doi: 10.1186/s13567-020-00802-z 32546223 PMC7298856

[29] Tønnesen MH, Penzhorn BL, Bryson NR, Stoltsz WH, Masibigiri T. Displacement of *Boophilus decoloratus* by *Boophilus microplus* in the Soutpansberg region, Limpopo Province, South Africa. *Exp Appl Acarol 2004 323*. 2004;32(3):199–208. doi: 10.1023/B:APPA.0000021789.44411.B5 15139085

[30] Shyma KP, Gupta JP, Singh V, Patel KK. In Vitro Detection of Acaricidal Resistance Status of *Rhipicephalus (Boophilus) microplus* against Commercial Preparation of Deltamethrin, Flumethrin, and Fipronil from North Gujarat, India. *J Parasitol Res*. 2015;2015. doi: 10.1155/2015/506586 26788362 PMC4691617

[31] Norval RAI, Perry BD, Meltzer MI, Kruska RL, Booth TH. Factors affecting the distributions of the ticks *Amblyomma hebraeum* and *A*. *variegatum* in Zimbabwe: implications of reduced acaricide usage. *Exp Appl Acarol*. 1994;18(7):383–407. doi: 10.1007/BF00051522 7628253

[32] Sungirai M, Abatih EN, Moyo DZ, De Clercq P, Madder M. Shifts in the distribution of ixodid ticks parasitizing cattle in Zimbabwe. *Med Vet Entomol*. 2017;31(1):78–87. doi: 10.1111/mve.12215 27935088

[33] Beati L, Patel J, Lucas-Williams H, et al. Phylogeography and demographic history of *Amblyomma variegatum* (Fabricius) (Acari: Ixodidae), the tropical bont tick. *Vector-Borne Zoonotic Dis*. 2012;12(6):514–525. doi: 10.1089/vbz.2011.0859 22448720 PMC3366099

[34] Jacquet S, Dupraz M, Stachurski F, et al. Genetic diversity of *Amblyomma variegatum* (Acari:Ixodidae), the main vector of *Ehrlichia ruminantium* in Indian Ocean Islands. *E-sove 2012 from Biol to Integr Control a Chang world Abstr B*. Published online 2012.

[35] Dahmani M, Davoust B, Sambou M, et al. Molecular investigation and phylogeny of species of the Anaplasmataceae infecting animals and ticks in Senegal. *Parasites and Vectors*. 2019;12(1):495. doi: 10.1186/s13071-019-3742-y 31640746 PMC6805679

[36] Sungirai M, Moyo DZ, De Clercq P, Madder M, Vanwambeke SO, De Clercq EM. Modelling the distribution of *Rhipicephalus microplus* and *R*. *decoloratus* in Zimbabwe. *Vet Parasitol Reg Stud Reports*. 2018;14:41–49. doi: 10.1016/j.vprsr.2018.08.006 31014735

[37] Magona JW, Walubengo J, Olaho-Mukani W, Jonsson NN, Welburn SW, Eisler MC. Spatial variation of tick abundance and seroconversion rates of indigenous cattle to *Anaplasma marginale*, *Babesia bigemina and Theileria parva* infections in Uganda. *Exp Appl Acarol*. 2011;55(2):203–213. doi: 10.1007/s10493-011-9456-2 21499913

[38] Murrell A, Campbell NJH, Barker SC. Phylogenetic analyses of the rhipicephaline ticks indicate that the genus *Rhipicephalus* is paraphyletic. *Mol Phylogenet Evol*. 2000;16(1):1–7. doi: 10.1006/mpev.2000.0762 10877935

[39] Beati L, Keirans JE. Analysis of the systematic relationships among ticks of the genera *Rhipicephalus* and *Boophilus* (Acari: Ixodidae) based on mitochondrial 12S ribosomal DNA gene sequences and morphological characters. *J Parasitol*. 2001;87(1):32–48. doi: 10.1645/0022-3395(2001)087[0032:AOTSRA]2.0.CO;2 11227901

[40] Mediannikov O, Diatta G, Fenollar F, Sokhna C, Trape JF, Raoult D. Tick-borne rickettsioses, neglected emerging diseases in rural Senegal. *PLoS Negl Trop Dis*. 2010;4(9). doi: 10.1371/journal.pntd.0000821 20856858 PMC2939048

[41] Dantas-Torres F, Latrofa MS, Annoscia G, Giannelli A, Parisi A, Otranto D. Morphological and genetic diversity of *Rhipicephalus sanguineu*s sensu lato from the New and Old Worlds. *Parasites and Vectors*. 2013;6(1). doi: 10.1186/1756-3305-6-213 23880226 PMC3735430

