# Supplementary figures and images for: Molecular survey of cattle ticks in Burundi: First report on the presence of the invasive Rhipicephalus microplus tick
Source: PLoS One. 2021 Dec 10;16(12):e0261218. doi: 10.1371/journal.pone.0261218 (PMC8664164; doi:10.1371/journal.pone.0261218)

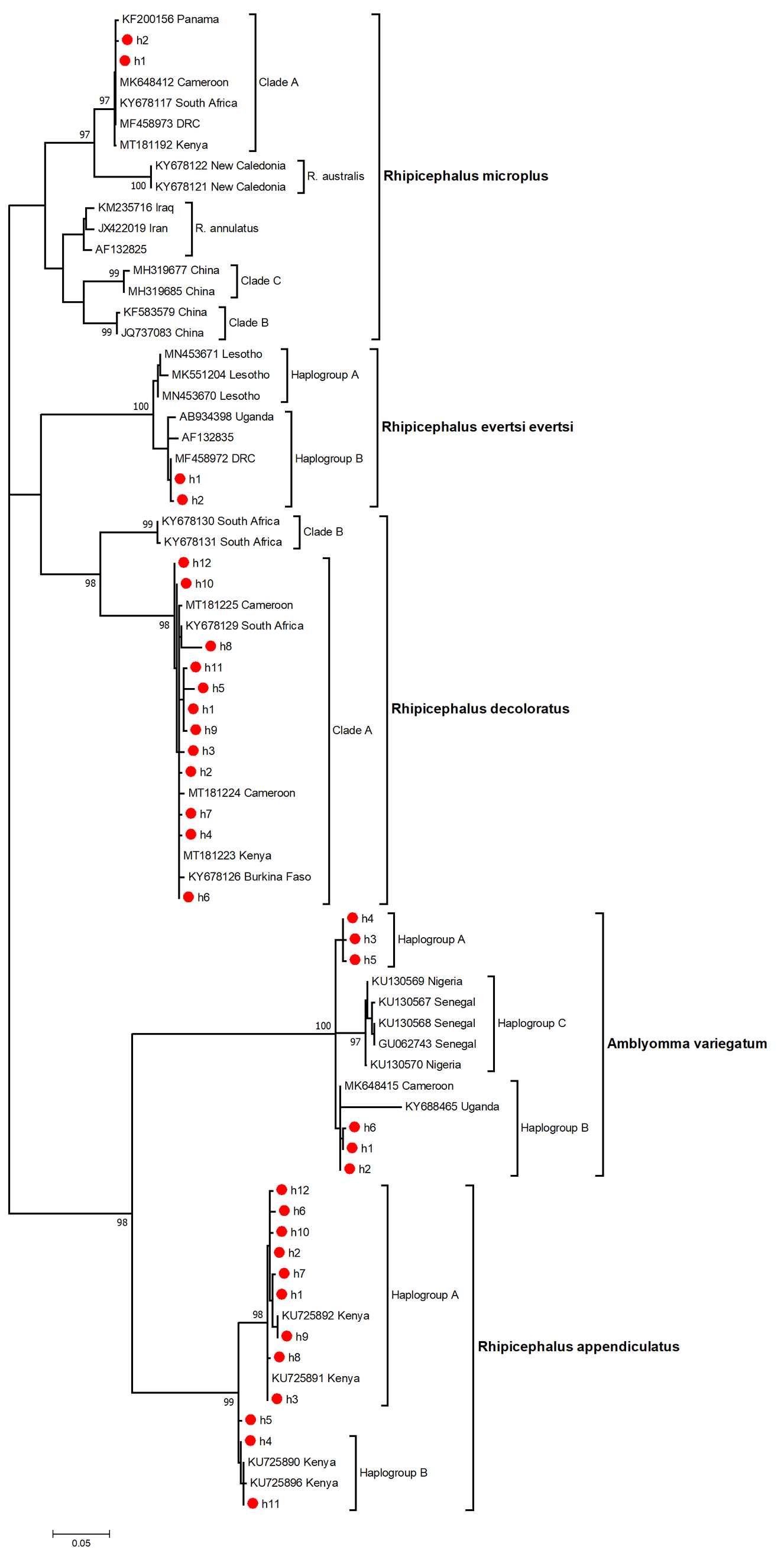

Supplement: S1 Fig — Haplotypes generated in the present study are highlighted with a red dot labeled with the letter h followed by a number (h1, hn). Evolutionary model used is Tamura 3-parameter (T92) with gamma distribution (+G = 4 categories). Clade with bootstrap value > 95% are shown. (TIF) [file pone.0261218.s001.tif]

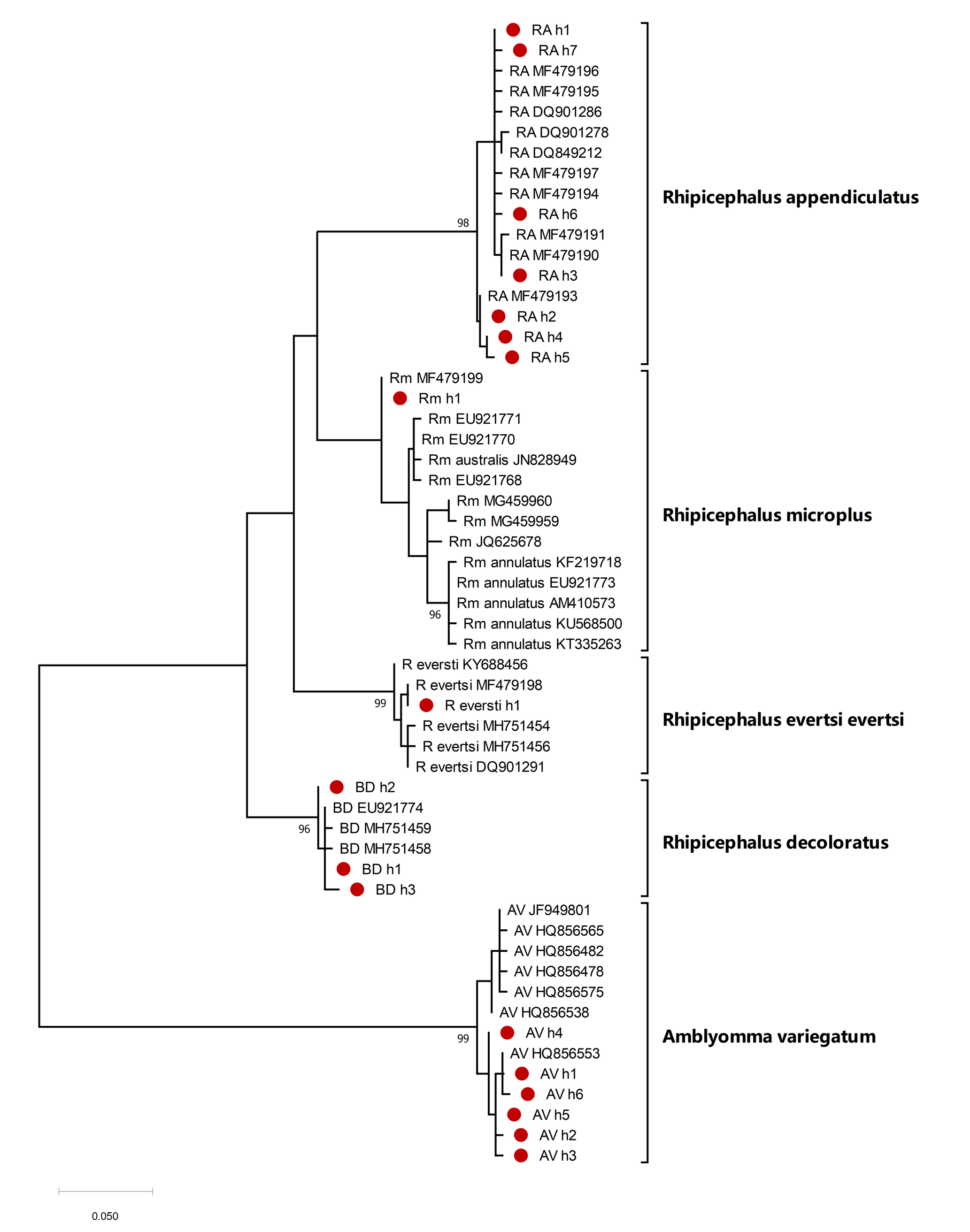

Supplement: S2 Fig — Haplotypes generated in the present study are highlighted with a red dot. Evolutionary model used is HKY with gamma distribution (+G = 4 categories). Clade with bootstrap value > 95% are shown. (TIF) [file pone.0261218.s002.tif]
