# Supplementary material for: Molecular survey of cattle ticks in Burundi: First report on the presence of the invasive Rhipicephalus microplus tick
Source: PLoS One. 2021 Dec 10;16(12):e0261218. doi: 10.1371/journal.pone.0261218 (PMC8664164; doi:10.1371/journal.pone.0261218)
Supplement: S1 Table — (DOCX) [file pone.0261218.s003.docx]

S1. Table. Nucleotide blast results of tick haplotypes

| Tick species | Gene locus | Haplotype | E value | identity % | Accession number | Origine | Reference |
| --- | --- | --- | --- | --- | --- | --- | --- |
| *Rhipicephalus microplus* | COI | H1 | 0 | 99.84 | MH513250.1 | French Guiana | [Binetruy F et al. 2019](https://www.ncbi.nlm.nih.gov/pubmed/?term=Binetruy%20F%5BAuthor%5D&cauthor=true&cauthor_uid=30224310) |
|  |  | H2 | 0 | 100 | MF363057.1 | Colombia | [Rivera-Páez FA, 2018](https://www.ncbi.nlm.nih.gov/pubmed/?term=Rivera-P%C3%A1ez%20FA%5BAuthor%5D&cauthor=true&cauthor_uid=29055642) |
|  | 12S | H1 | 0 | 100 | MK234703.1 | India | Direct Submission |
| *Rhipicephalus appendiculatus* | COI | H1 | 0 | 100 | KX276928.1 | Kenya | Kanduma et al. 2016 |
|  |  | H2 | 0 | 100 | MF458945.1 | Tanzania | Amzati et al. 2018 |
|  |  | H3 | 0 | 100 | KU725891 | Kenya | Kanduma et al. 2016 |
|  |  | H4 | 0 | 100 | MF458915.1 | DRC | Amzati et al. 2018 |
|  |  | H5 | 0 | 99.44 | MT181227.1 | Kenya | Kanduma et al. 2016 |
|  |  | H6 | 0 | 99.63 | MF458971.1 | DRC | Amzati et al. 2018 |
|  |  | H7 | 0 | 99.81 | KX276899.1 | Kenya | Kanduma et al. 2016 |
|  |  | H8 | 0 | 100 | MF458961.1 | DRC | Amzati et al. 2018 |
|  |  | H9 | 0 | 100 | MF133499.1 | DRC | Amzati et al. 2018 |
|  |  | H10 | 0 | 99.81 | KX276942.1 | Zambia | Kanduma et al. 2016 |
|  |  | H11 | 0 | 100 | KC503257 | Zimbabwe | Burger et al. 2014 |
|  |  | H12 | 0 | 99.81 | KX276926 | Kenya | Kanduma et al. 2016 |
|  | 12S | H1 | 0 | 100 | DQ849209.1 | Zambia | Direct Submission |
|  |  | H2 | 7e-180 | 99.45 | MH751457.1 | South Africa | Guo,H. et al.2019 |
|  |  | H3 | 7e-180 | 99.72 | DQ849209.1 | Zambia | Direct Submission |
|  |  | H4 | 3e-178 | 99.17 | MH751457.1 | South Africa | Guo,H. et al.2019 |
|  |  | H5 | 1e-176 | 99.16 | DQ849203.1 | Zambia | Direct Submission |
|  |  | H6 | 3e-172 | 99.42 | MF479186.1 | DRC | Amzati et al. 2018 |
|  |  | H7 | 7e-180 | 99.72 | DQ849209.1 | Zambia | Direct Submission |
| *Rhipicephalus decoloratus* | Cox1 | H1 | 0 | 99.66 | MT181223.1 | Kenya | Direct Submission |
|  |  | H2 | 0 | 99.83 | MK648414.1 | Cameroon | Direct Submission |
|  |  | H3 | 0 | 99.66 | KY678128.1 | South Africa | Duron,O. et al. 2017 |
|  |  | H4 | 0 | 99.83 | KY678128.1 | South Africa | Duron,O. et al. 2017 |
|  |  | H5 | 0 | 98.98 | MT181223.1 | Kenya | Direct Submission |
|  |  | H6 | 0 | 100 | MK648414.1 | Cameroon | Direct Submission |
|  |  | H7 | 0 | 99.83 | MT181223.1 | Kenya | Direct Submission |
|  |  | H8 | 0 | 98.64 | KY678129.1 | South Africa | Duron,O. et al. 2017 |
|  |  | H9 | 0 | 99.49 | MT181223.1 | Kenya | Direct Submission |
|  |  | H10 | 0 | 99.83 | MK648413.1 | Cameroon | Direct Submission |
|  |  | H11 | 0 | 99.49 | MT181223.1 | Kenya | Direct Submission |
|  |  | H12 | 0 | 99.66 | MT181222.1 | Kenya | Direct Submission |
|  | 12S | H1 | 3e-171 | 100 | KF569940.1 | Mali | McCoy,B. et al. 2013 |
|  |  | H2 | 1e-169 | 99.71 | KF569940.1 | Mali | McCoy,B. et al. 2013 |
|  |  | H3 | 2e-167 | 99.41 | AF150044.1 | Zimbabwe | Beati,L. 1999 |
| *Amblyoma variegatum* | Cox1 | H1 | 0 | 99.83 | MK648415.1 | Cameroon | Silatsa,B.A.et al.2019 |
|  |  | H2 | 0 | 100 | MK648415.1 | Cameroon | Silatsa,B.A.et al.2019 |
|  |  | H3 | 0 | 96.74 | KU568507.1 | Guinea-Bissau | Zuquete,S.T.et al. 2016 |
|  |  | H4 | 0 | 99.14 | MK648415.1 | Cameroon | Silatsa,B.A.et al.2019 |
|  |  | H5 | 0 | 98.97 | MK648415.1 | Cameroon | Silatsa,B.A.et al.2019 |
|  |  | H6 | 0 | 99.66 | MK648415.1 | Cameroon | Silatsa,B.A.et al.2019 |
|  | 12S | H1 | 1e-163 | 100 | HQ856466.1 | Burkina Faso | Beati,L. et al.2012 |
|  |  | H2 | 2e-162 | 99.69 | HQ856491.1 | Kenya | Beati,L. et al.2012 |
|  |  | H3 | 2e-160 | 99.39 | HQ856513.1 | Kenya | Beati,L. et al.2012 |
|  |  | H4 | 2e-162 | 99.69 | HQ856491.1 | Kenya | Beati,L. et al.2012 |
|  |  | H5 | 4e-164 | 100 | HQ856491.1 | Kenya | Beati,L. et al.2012 |
|  |  | H6 | 5e-162 | 99.69 | HQ856466.1 | Ethiopia | Beati,L. et al.2012 |
| Rhipicaphalus evertsi evertsi | Cox1 | H1 | 0 | 98.31 | AB934398.1 | Uganda | Direct Submission |
|  |  | H2 | 0 | 98.16 | AB934398.1 | Uganda | Direct Submission |
|  | 12S | H1 | 3e-159 | 100 | MF479198.1 | DRC | Amzati et al. 2018 |
