# Supplementary material for: Molecular survey of cattle ticks in Burundi: First report on the presence of the invasive Rhipicephalus microplus tick
Source: PLoS One. 2021 Dec 10;16(12):e0261218. doi: 10.1371/journal.pone.0261218 (PMC8664164; doi:10.1371/journal.pone.0261218)
Supplement: S2 Table — (DOCX) [file pone.0261218.s004.docx]

S2 Table. Mitochondrial nucleotide and haplotype diversity of infesting cattle ticks in Burundi

|  | N | L | C | S | H | Hd ± SD | Theta  (per site) | Theta (sequence) |
| --- | --- | --- | --- | --- | --- | --- | --- | --- |
| *R. microplus* |  |  |  |  |  |  |  |  |
| Cox1 | 40 | 622 | 0.998 | 1 | 2 | 0.050±0.047 | 0.00038 | 0.235 |
| 12S | 30 | 361 | - | 0 | 1 | 0 | 0 | 0 |
| *R. appendiculatus* |  |  |  |  |  |  |  |  |
| Cox1 | 106 | 536 | 0.963 | 20 | 12 | 0.737±0.035 | 0.00713 | 3.820 |
| 12S | 101 | 361 | 0.981 | 7 | 7 | 0.378±0.054 | 0.00374 | 1.349 |
| *R. decoloratus* |  |  |  |  |  |  |  |  |
| Cox1 | 43 | 588 | 0.963 | 22 | 12 | 0.848±0.032 | 0.00865 | 5.085 |
| 12S | 40 | 339 | 0.991 | 3 | 3 | 0.099±0.064 | 0.00208 | 0.705 |
| *A. variegatum* |  |  |  |  |  |  |  |  |
| Cox1 | 33 | 583 | 0.985 | 9 | 6 | 0.629±0.078 | 0.00380 | 2.218 |
| 12S | 33 | 332 | 0.982 | 6 | 6 | 0.424±0.105 | 0.00445 | 1.478 |
| *R. eversti evertsi* |  |  |  |  |  |  |  |  |
| Cox1 | 6 | 648 | 0.998 | 1 | 2 | 0.333±0.215 | 0.00068 | 0.438 |
| 12S | 6 | 317 | - | 0 | 1 | 0 | 0 | 0 |

N:Sample size, S: Number of polymorphic sites; H: Number of Haplotypes; Haplotype (gene) diversity, SD: standard deviation, L: Net number of analyzed sites, C: Sequence conservation, Theta: Watterson estimator(from S).
